# Effectiveness and safety of repetitive transcranial magnetic stimulation (rTMS) on aphasia in cerebrovascular accident patients: Protocol of a systematic review and meta-analysis

**DOI:** 10.1097/MD.0000000000018561

**Published:** 2019-12-27

**Authors:** Yaling Zheng, Dongling Zhong, Yijie Huang, Mingxing He, Qiwei Xiao, Rongjiang Jin, Juan Li

**Affiliations:** a School of Health Preservation and Rehabilitation; b School of Acupuncture, Moxibustion and Tuina/The Third Affiliated Hospital, Chengdu University of Traditional Chinese Medicine, Chengdu, Sichuan, China.

**Keywords:** aphasia, cerebrovascular accident, effectiveness, safety, rTMS, protocol, meta-analysis, systematic review

## Abstract

**Background:**

Repetitive transcranial magnetic stimulation (rTMS), a non-invasive brain stimulation approach, might be a promising technique in the management of aphasia after cerebrovascular accidents (CVA). This protocol of systematic review (SR) aims to investigate the effectiveness and safety of rTMS in patients with aphasia after CVA.

**Methods:**

The following databases will be searched: PubMed, EMBASE, the Cochrane Central Register of Controlled Trials (CENTRAL), China National Knowledge infrastructure (CNKI), Technology Periodical Database (VIP), WanFang Data, and China Biology Medicine (CBM) from inception to August 2019. Randomized controlled trials (RCTs) investigating the effectiveness and safety of rTMS for aphasia patients after CVA will be included. Primary outcome will include Boston Diagnostic Aphasia Examination (BDAE). Secondary outcomes will include Aphasia Battery of Chinese (ABC), Aachen Aphasia Test (AAT), Aphasia Quotient (AQ), the Western Aphasia Battery (WAB), Standard Language Test of Aphasia (SLTA), Aphasia Severity Rating Scale (ASRS), Concise China Aphasia Test Scale (CCAT), Amsterdam-Nijmegen Everyday Language Test (ANELT), or other related outcomes. Adverse events such as headache, tinnitus, anxiety, fatigue, or epileptic seizure will be considered as safety measurement. Studies screening, data extraction, and methodological quality assessment will be performed independently by two reviewers. Meta-analysis will be conducted with Review Manager 5.3 software and R software 3.6.1.

**Results:**

This study will provide a high-quality synthesis of RCTs on the effectiveness and safety of rTMS as an adjuvant therapy in the treatment of aphasia.

**Conclusion:**

The conclusion of this study will help clinicians and patients with aphasia after CVA to make decision.

**Ethics and dissemination:**

No privacy health information will be collected, thus formal ethics approval is not required. The findings of this SR will be submitted to a peer-reviewed journal.

**PROSPERO registration number:**

CRD42019144587.

## Abstract 2

Supplemental Digital Content is available in the text

## 1 Introduction

Aphasia is a serious acquired communication disorder syndrome resulted from damage to the brain language functional areas and related language networks.[^1^,^2^] Although other causes such as brain tumor, serious infection, or head trauma can also cause aphasia,[^3^,^4^] aphasia happened mostly in patients with cerebrovascular accident (CVA).[^5^,^6^] Epidemiological data from China show that there are over 2 million new CVA cases annually.^[7]^ About 30% of CVA patients suffered from aphasia,[^1^,^8^] we speculate that about 600,000 new aphasia CVA patients will be added in china each year.^[9]^ And 12% of CVA survivors are still aphasic at six months.^[10]^ In the United States, at least 1 million people suffer from aphasia caused by CVA.^[11]^ As one of the most devastating symptoms in CVA survivors,[^12^,^13^] aphasia often leads to a range of communication deficits, including language comprehension, language expression, reading, writing, attention, memory, and other cognitive domains.[^14^,^15^] Aphasia can also cause a series of functional disorders, emotional disorders, social participation disorders, and limitations on activities of daily life that greatly reduce the quality of life.[^16^,^17^,^18^,^19^,^20^] Besides, patients with aphasia after CVA have a serious financial burden on families and society, and their care costs are approximately $7100 higher than those of non-aphasia patients.^[12]^ Due to the high incidence and risk, aphasia rehabilitation is listed as one of the top 10 research priorities related to the life after CVA.^[21]^ At present, the main treatment for aphasia caused by CVA is traditional language behavior training, which can improve the language communication ability and quality of life to a certain extent,^[10]^ but the recovery degree of communication ability is still limited.[^11^,^22^] Recently, non-invasive cortical stimulation gets great attention as treatment for aphasia patients with CVA.^[23]^

Repetitive transcranial magnetic stimulation (rTMS) is a non-invasive and painless method of altering the excitability of the cerebral cortex^[24]^ by inducing or enhancing neuroplasticity in brain.[^25^,^26^] Growing evidence indicates that rTMS has beneficial effects for patients with aphasia caused by CVA.[^26^,^27^,^28^,^29^,^30^,^31^] Stimulating the right hemisphere of aphasia patients after CVA with rTMS can improve language functions such as content, fluency, aphasia quotient, dysarthria, repetition, naming performance, expressive language, auditory comprehension, and command comprehension.[^26^,^27^,^32^]

There are currently three systematic reviews (SRs) and meta-analyses on the effectiveness and safety of rTMS in the treatment of aphasia caused by CVA,[^33^,^34^,^35^] the most recent one was published in 2015.^[34]^ Besides, all of them are of poor methodological quality. Wang et al^[33]^ included three duplicates. Li et al^[34]^ did not use comprehensive literature search strategy. Ren et al^[35]^ did not report in accordance with Preferred Reporting Items for Systematic reviews and Meta-Analysis (PRISMA). None of these SRs had registered in Prospective Register of Systematic Reviews (PROSPERO) and reported study protocols in advance, so the results may be biased. Overall, there is a lack of supportive evidence on the effectiveness and safety of rTMS for aphasia after cerebrovascular accident. Therefore, our SR aims to conduct a SR and meta-analysis of randomized controlled trials (RCTs) on the effectiveness and safety of rTMS as an adjuvant therapy in the treatment of aphasia. This protocol will be reported according with the Preferred Reporting Items for Systematic Review and Meta-Analysis Protocols (PRISMA-P).

## 2 Methods and analysis

### 2.1 Study registration

Our SR has registered on PROSPERO (registration number CRD: 42019144587), and will be reported adhere to the PRISMA.^[36]^

### 2.2 Inclusion criteria

#### 2.2.1 Types of studies and publications

We will only include RCTs using rTMS for CVA patients with aphasia, which are published in Chinese and English.

#### 2.2.2 Types of participants

We will include right-handed adults (≥18 years old) with first stroke who are diagnosed with CVA by The Fourth National Cerebrovascular Disease Conference in 1995,^[37]^ and confirmed by imaging examination (brain CT or MRI). The diagnosis of aphasia will be judged by the aphasia rating scales, such as the Boston Diagnostic Aphasia Examination (BDAE) and Aphasia Battery of Chinese (ABC). Gender, ethnicity, education level, CVA location, disease duration, type of aphasia, and severity of aphasia (assessed according to Aphasia Severity Rating Scale (ASRS) will not be restricted.

#### 2.2.3 Types of interventions

All groups will receive the standard treatment (drug therapy, conventional physical exercises, or speech training). Besides, the experimental group will receive rTMS and the control group will receive sham rTMS or other active treatments.

#### 2.2.4 Outcome measurements

Primary outcome will include BDAE. Secondary outcomes will include ABC, Aachen Aphasia Test (AAT), Aphasia Quotient (AQ), the Western Aphasia Battery (WAB), Standard Language Test of Aphasia (SLTA), ASRS, Concise China Aphasia Test Scale (CCAT), Amsterdam-Nijmegen Everyday Language Test (ANELT), or other related outcomes. Adverse events such as headache, tinnitus, anxiety, fatigue, or epileptic seizure will be considered as safety measurement.

### 2.3 Exclusion criteria

(1) Aphasia caused by brain tumor, serious infection, head trauma, or other causes;
(2) Repeated publications or documents that data cannot be extracted;
(3) Full text cannot be obtained through various approaches.

### 2.4 Database and search

We will search PubMed, EMBASE, the Cochrane Central Register of Controlled Trials (CENTRAL), China National Knowledge infrastructure (CNKI), Technology Periodical Database (VIP), and WanFang Data and China Biology Medicine (CBM) from inception to August 2019, using a combination of relevant keywords and subject terms. The following terms will be searched: rTMS, repetitive transcranial magnetic stimulation, cerebrovascular accident, stroke, aphasia, RCT. The full search strategy of PubMed is provided in Appendix 1 and similar strategies will be applied to the other electronic databases. We will additionally search the grey literature and reference lists of identified articles to avoid missing eligible RCTs.

### 2.5 Studies selection

We will select studies for inclusion in two stages. In the first stage, we will screen the titles and abstracts for potentially relevant papers. In the second stage, two reviewers (Yaling Zheng and Dongling Zhong) will independently screen and assess the full-text according to prespecified inclusion criteria. Disagreements will be resolved by discussion or consultation with an experienced reviewer (Rongjiang Jin). Details of the entire selection procedure will be shown in flow chart (Appendix 2).

### 2.6 Data extraction

Two reviewers will independently (Yaling Zheng and Dongling Zhong) extract data using a predefined data extraction template. Any discrepancies will be resolved by the third reviewer (Juan Li). Following items will be included:

(1) Study characteristics: first author, title, journal, year of publication, country, funding source, etc.
(2) Participant characteristics: sample size, age, gender, disease duration, comorbidity, etc.
(3) Intervention characteristics: protocol of intervention (stimulus frequency, intensity, pulse, target area, duration, etc.), protocol of comparisons (type, frequency, dose, or duration).
(4) Trial characteristics: design, method of randomization, allocation concealment, blinding (subjects, therapists, and assessors), etc.
(5) Outcomes: primary outcome, secondary outcome, main conclusions, adverse events, etc.

The original authors will be contacted in case of missing data. As for discrepancy, two reviewers will resolve through team discussion.

### 2.7 Methodological quality assessment

Two reviewers (Yijie Huang and Mingxing He) will independently use the Physiotherapy Evidence Database (PEDro) scale as methodological criteria. This scale has 11 criteria, each criteria answers “yes” and “no”, with a total score of 10 (scores of 8–10 represent a good-quality study; scores of 6 and 7 represent a fair-quality study; and scores of 5 or lower represent a low-quality study). In case of disagreements, a third reviewer (Qiwei Xiao) will be involved.

### 2.8 Data analysis

Analyses for all outcomes will be done on an intention-to-treat basis. Statistical analyses will be conducted using RevMan 5.3 software and R software 3.6.1. We will use mean difference to analyze the various aphasia assessment outcomes such as BADE, ABC, WAB, SLTA, CCAT, and ANELT. And the standardized mean difference (SMD) will be used to analyze ASRS. The uncertainly will be expressed with 95% confidence intervals (95%CI). Heterogeneity of included studies will be evaluated by *χ* ^2^ test, and the test level will set as *α* = 0.1. If *P* > .1 and *I* ^2^ < 50%, indicating good homogeneity among the results, a fixed effect model will be used for meta-analysis. If *P* ≤ .1 and/or *I* ^2^ ≥ 50%, there may be statistical heterogeneity among the results. A meta-analysis will then be performed using a random effect model. Heterogeneity will be further explored using subgroup or sensitivity analysis. Results will be described qualitatively in the text when meta-analysis is not possible.

#### 2.8.1 Subgroup analysis

We intend to perform subgroup analysis by type of CVA (cerebral hemorrhage or cerebral infarction), stimulation frequency [low (≤1 Hz) versus high (≥5 Hz)], type of aphasia (non-fluent aphasia or fluent aphasia), target area (frontal area or broca area), and different comparisons (rTMS therapy versus sham rTMS, conventional treatment or other active therapies).

#### 2.8.2 Sensitivity analysis

We will carry out sensitivity analyses on the following factors to assess the impact of study quality: concealed allocation, outcomes assessor blinding, and drop outs.

#### 2.8.3 Publication bias

Publication bias will be evaluated with the funnel plot of asymmetry and the Orwin fail-safe N approach. If the funnel plot is asymmetrical, indicating that publication bias exists, the Egger test will be used to evaluate whether the amount of asymmetry is significant. In addition, studies that demonstrated a lack of benefit may not have been published or submitted for publication. Therefore, we will use the Orwin fail-safe *N* test to estimate the number of missing studies. If *N* > 5k + 10, there will be no publication bias.

### 2.9 Quality of evidence

The quality of the evidence of each outcome will be independently assessed by two reviewers (Yaling Zheng and Juan Li) with GRADEpro V.3 software and rated as high, moderate, low, or very low level according to the Grading of Recommendations Assessment, Development and Evaluation system (GRADE).^[38]^

### 2.10 Ethics and dissemination

No privacy health information will be collected, thus formal ethics approval is not required. The findings of this SR will be submitted to a peer-reviewed journal.

## 3 Discussion

Recently, rTMS has gained increasing popularity in functional rehabilitation among patients with CVA. Evidence suggests that rTMS shows beneficial effects on speech disorder of aphasia patients with CVA. The theoretical basis of rTMS for treatment of aphasia with CVA mainly relies on the theory of “transcallosal inhibition”.^[39]^ Under normal physiological conditions, the human left and right cerebral hemispheres are mutually inhibited by the corpus callosum and are in a state of dynamic equilibrium.^[40]^ When the dominant hemisphere of the language is damaged, the inhibition of the right hemisphere from the left hemisphere is weakened, and the excitability of the right hemisphere is increased, which in turn increases the inhibition to the left hemisphere, resulting in a further decrease in the excitability of the previously damaged left hemisphere. Studies have confirmed that rTMS can restore the equilibrium physiological state of both hemispheres by adjusting the stimulation parameters. Low-frequency rTMS (≤1 Hz) is commonly used to decrease cortical excitability, while high-frequency rTMS (≥5 Hz) is applied to facilitate it.[^39^,^40^,^41^] Therefore, rTMS can stimulate the left hemisphere at high frequency or inhibit the right hemisphere at low frequency, thus promoting the recovery of language function.[^41^,^42^] In the three previously published meta-analyses in the treatment of aphasia with rTMS, both the number and sample size of included studies were small, which are prone to false positives. In addition, the control groups included in the literature were mostly sham control, rather than other active rehabilitation therapies, which is not conducive to the clinical selection of the best scheme for the treatment of aphasia. Most importantly, a growing number of RCTs on rTMS for the treatment of post-stroke aphasia were published between 2014 and 2019.[^2^,^43^,^44^,^45^] Therefore, we plan to conduct a SR and meta-analysis to assess the effectiveness and safety of rTMS in CVA patients with aphasia, hoping our results may help clinicians and patients make clinical decisions.

## 4 Strengths and limitations

This protocol has been registered in PROSPERO. The SR will be conducted in strict accordance with the standards of the AMSTAR 2.0 and reported according to PRISMA. However, there is still a potential limitation. The language will be limited in Chinese and English, and the literature published in other languages is not included.

## Author contributions

**Conceptualization:** Yaling Zheng, Dongling Zhong.

**Methodology:** Yaling Zheng, Dongling Zhong, Yijie Huang, Mingxing He, Qiwei Xiao.

**Supervision:** Rongjiang Jin, Juan Li.

**Writing – original draft:** Yaling Zheng, Dongling Zhong.

**Writing – review & editing:** Yaling Zheng, Dongling Zhong.

## Supplementary Material

Supplemental Digital Content

## Supplementary Material

Supplemental Digital Content
