# Supplementary material for: Effectiveness and safety of repetitive transcranial magnetic stimulation (rTMS) on aphasia in cerebrovascular accident patients: Protocol of a systematic review and meta-analysis
Source: Medicine (Baltimore). 2019 Dec 27;98(52):e18561. doi: 10.1097/MD.0000000000018561 (PMC6946413; doi:10.1097/MD.0000000000018561)
Supplement: Supplemental Digital Content [file medi-98-e18561-s001.docx]

**Appendix 1: PubMed search strategy**

#1　Stroke[Mesh]

#2　Stroke[Title/Abstract]) OR Cerebrovascular Accident[Title/Abstract]) OR CVA (cerebrovascular accident)[Title/Abstract]) OR Apoplexy[Title/Abstract]) OR Brain vascular accident[Title/Abstract]

#3　#1 OR #2

#4　Aphasia[Mesh]

#5　Aphasia[Title/Abstract]) OR Logagnosia[Title/Abstract]) OR Alogia[Title/Abstract]) OR Anepia [Title/Abstract]) OR Dysphasia[Title/Abstract]) OR Language Disorders [Title/Abstract]) OR Anomia [Title/Abstract]) OR Linguistic Disorders[Title/Abstract]

#6　#4 OR #5

#7　Transcranial Magnetic Stimulation"[Mesh]

#8　Transcranial Magnetic Stimulation[Title/Abstract]) OR Repetitive Transcranial Magnetic Stimulation,[Title/Abstract]) OR TMS[Title/Abstract]) OR rTMS[Title/Abstract]

#9　#7 OR #8

#10　Randomized Controlled Trial[Publication Type]

#11　Randomized Controlled Trials[Title/Abstract] OR Clinical Trials, Randomized [Title/Abstract] OR Trials, Randomized Clinical[Title/Abstract]OR Randomized [Title/Abstract]

#12　#10 OR #11

#13　#3 AND #6 AND #9 AND #12
