# Supplementary material for: Effectiveness and safety of repetitive transcranial magnetic stimulation (rTMS) on aphasia in cerebrovascular accident patients: Protocol of a systematic review and meta-analysis
Source: Medicine (Baltimore). 2019 Dec 27;98(52):e18561. doi: 10.1097/MD.0000000000018561 (PMC6946413; doi:10.1097/MD.0000000000018561)
Supplement: Supplemental Digital Content [file medi-98-e18561-s002.docx]

**Appendix 2**

**Identification**

**Included**

**Screening**

**Eligibility**

Records identified through database searching(n=)

Additional records identified through other sources(n=)

Records after duplicates removed(n=)

Records screened(n=)

Records excluded

Not related to (n=)

Animal(n=)

Reviews(n=)

Not RCT(n=)

Case studies(n=)

Full-text articles assessed for eligibility(n=)

Studies included in qualitative synthesis (n=)

Full-text articles excluded with reasons(n=)

Studies included in quantitative synthesis (meta-analysis)(n=)
